# Are Circulating Tumor Cells (CTCs) Ready for Clinical Use in Breast Cancer? An Overview of Completed and Ongoing Trials Using CTCs for Clinical Treatment Decisions

**DOI:** 10.3390/cells8111412

**Published:** 2019-11-08

**Authors:** Fabienne Schochter, Thomas W. P. Friedl, Amelie deGregorio, Sabrina Krause, Jens Huober, Brigitte Rack, Wolfgang Janni

**Affiliations:** Department of Obstetrics and Gynecology, University Hospital Ulm, University of Ulm, 89075 Ulm, Germany; Amelie.deGregorio@uniklinik-ulm.de (A.d.G.); sabrina.krause@uniklinik-ulm.de (S.K.); jens.huober@uniklinik-ulm.de (J.H.); brigitte.rack@uniklinik-ulm.de (B.R.); wolfgang.janni@uniklinik-ulm.de (W.J.)

**Keywords:** circulating tumor cells (CTCs), clinical trials, breast cancer, CTC-based treatment decisions

## Abstract

In recent years, breast cancer treatment has become increasingly individualized. Circulating tumor cells (CTCs) have the potential to move personalized medicine another step forward. The prognostic relevance of CTCs has already been proven both in early and metastatic breast cancer. In addition, there is evidence that changes in CTC numbers during the course of therapy can predict treatment response. Thus, CTCs are a suitable tool for repeated treatment monitoring through noninvasive liquid biopsy. The next step is to evaluate how this information can be used for clinical decision making with regard to the extension, modification, or abandonment of a treatment regimen. This review will summarize the completed and ongoing clinical trials using CTC number or phenotype for treatment decisions. Based on current knowledge, CTCs can be regarded as a useful prognostic and predictive marker that is well suited for both risk stratification and treatment monitoring in breast cancer patients. However, there is still the need to provide sufficient and unequivocal evidence for whether CTCs may indeed be used to guide treatment decisions in everyday clinical practice. The results of the ongoing trials described in this review are eagerly awaited to answer these important questions.

## 1. Introduction

In recent years, breast cancer treatment has become increasingly individualized. New targeted therapies have improved survival for many patients. With the aim of becoming even more patient-specific, circulating tumor cells (CTCs) are an interesting topic in translational oncology. CTCs represent rare cancer cells in the peripheral blood that have disseminated from the primary tumor (or metastatic sites) and play an important role in tumor progression and the formation of (new) metastases. Several recent reviews describe the biology of CTCs and discuss both their potential of being used as a prognostic and/or predictive marker and the challenges involved in incorporating CTCs in clinical practice [1,2].

CTCs have already proven their prognostic relevance in early breast cancer (EBC) and metastatic breast cancer (MBC) [3,4,5]. In addition, the SUCCESS-A and the ECOG-ACRIN study E5103 showed that the detection of persisting CTCs two years and even five years after (neo)adjuvant chemotherapy is related to an increased risk of recurrence [6,7,8]. In a multivariate analysis presented by Sparano 2018, CTC-positivity assessed after five years was the strongest predictor of late disease recurrence in patients with hormone receptor-positive breast cancer that had no signs of disease recurrence in the first five years after primary diagnosis [8].

If CTC-positivity predicts a worse clinical outcome [9] and CTC dynamics is a predictor of therapy response [10], the question remains whether this information can be used for forming treatment decisions. Trials are attempting to find a way to answer this challenging question using either CTC number or CTC phenotype as a criterion for therapy decisions. This article gives an overview of the current status (see Table 1) and—if available—results of all clinical breast cancer trials that involve interventions based on CTC number or phenotype.

## 2. Material and Methods

On the database site ClinicalTrials.gov, 59 studies were retrieved by searching using the keywords “circulating tumor cells” and “breast cancer” at the time the search was performed (end of March 2019). Overall, 26 trials were initiated in Europe, 20 in the United States, 11 in Asian countries, and 2 in Brazil. In total, 31 interventional trials are registered, from which 10 are either recruiting or active but not recruiting. However, only 7 trials use either the presence, amount, or phenotype of CTCs in determining an intervention for the chosen treatment. One is an adjuvant trial, while the others describe trials in a metastatic setting. In the following, we will give an overview of these studies.

All clinical trials used the only FDA-cleared and current gold standard method for the enrichment and detection of CTCs, the CELLSEARCH^®^ system (Menarini Silicon Biosystems, Bologna, Italy), which has been described in detail by Park Y et al. [11]. Briefly, the first step during analysis is the immunomagnetic enrichment of EpCAM-positive cells using antibody-coated ferrofluid nanoparticles. The EpCAM-enriched cells are then stained with antibodies specific for cytokeratins 8, 18, and 19 (epithelial cell markers) and for CD45 (leukocyte marker), as well as with the fluorescent nucleic acid dye 4,6-diamidino-2-phenylindole dihydrochloride (DAPI) for labeling of cell nuclei. CTCs are identified and counted using a semiautomated fluorescence-based microscope system that generates images of the stained cells, with CTCs being defined as cytokeratin-positive nucleated cells that lack *CD45* expression.

## 3. CTC-Based Clinical Trials in EBC

### Treat-CTC

The TREAT-CTC study (NCT01548677) is the only study in the (neo)adjuvant setting in which treatment decisions are based on the presence of CTCs. This trial tried to address the question of an additional treatment possibility to eliminate CTCs persistent after (neo)adjuvant chemotherapy. A total of 1317 patients with HER2-negative EBC were screened for CTCs after completion of (neo)adjuvant chemotherapy and patients with at least 1 CTC/15 mL blood were randomized to either an additional treatment with trastuzumab (6 cycles of trastuzumab i.v.) or observation. In 95 (7.2%) of the patients, CTCs could be detected; 31 patients were randomized to trastuzumab treatment, while 32 patients were randomized to the observational control arm. The CTC-positivity rate was similar, and not significantly different in both arms after 18 weeks of treatment (17.2% vs. 13.8%); furthermore, no difference in disease-free survival could be seen [12]. Following the recommendation of the independent Data Monitoring Committee to stop the trial for futility, study recruitment was not continued after the first interim analysis. A possible explanation for this negative result might be that, while the HER2 status of CTCs was determined, HER2-positivity of CTCs was not required for study inclusion. In the majority of the patients (76%), the detected CTCs were HER2-negative. This is in accordance with the results of the NSABP-B47 trial that failed to show improved disease-free survival if trastuzumab is added to chemotherapy in patients with HER2-low (IHC 1+ or 2+ staining intensity) breast cancer [13]. Thus, both the NSABP B47 and the Treat CTC trial failed to confirm the hypothesis that women with early breast cancer showing low *HER2* expression might benefit from treatment with trastuzumab following adjuvant chemotherapy. Taken together, these results suggest that the failure of the Treat CTC trial was due to choosing an inappropriate treatment intervention for the targeted patient population rather than indicating a general failure of the concept of CTC-based intervention decisions.

## 4. CTC-Based Clinical Trials in MBC

In the MBC setting, trials that are based on CTC number usually use the cutoff of ≥5 CTCs which, initially, was not meant to be used for treatment decisions but was presented as a tool to separate the patients into two groups with different survival prospects. A recent retrospective pooled analysis including 2436 MBC patients confirmed the utility of the cutoff of ≥5 CTCs for risk stratification, as MBC patients could be separated into categories of either stage IV indolent (<5 CTCs) or stage IV aggressive (≥5 CTCs) with significantly longer overall survival in the group with <5 CTCs independently of clinical and molecular variables [14]. 

### 4.1. SWOG S0500

Since the first knowledge that a high count of CTCs predicts a worse clinical outcome and that changes in CTCs reflect therapy response, the question has been raised of whether MBC patients can be monitored and treated based on CTC dynamics. The first clinical phase III trial to investigate this hypothesis was initiated by the Southwest Oncology Group (SWOG). The SO500 study (NCT00382018) included 595 patients with MBC scheduled for first-line chemotherapy in the advanced setting. Before the start of first-line chemotherapy, patients were tested for CTCs. If patients did not have an increased CTC count (defined as less than 5 CTCs per 7.5 mL blood) at baseline (n = 276), they were treated according to physician’s choice and no additional blood draws or interventions were performed. A total of 319 patients had an elevated CTC count (defined as five or more CTCs per 7.5 mL blood) before the start of first-line chemotherapy. A total of 288 of these high-risk patients were re-tested after the first cycle of first-line therapy, which was approximately 22 days after the first chemotherapy administration. Patients with a follow-up CTC count of less than 5 CTCs per 7.5 mL blood (n = 165) continued on the initially chosen chemotherapy regimen until progression. If the follow-up CTC level was persistently high (i.e., the CTC count remained at a level of five or more CTCs per 7.5 mL blood), the patients (n = 123) were included in the interventional part and randomly assigned to either continue the treatment until clinical and/or radiographic evidence of progression (n = 64) or switch early to another chemotherapy of the physician’s choice (n = 59). The results showed no significant improvement in survival for an early change in treatment regime (median progression-free survival (PFS) 4.6 vs. 3.5 months, HR = 0.92; 95%CI = 0.64–1.32; median overall survival (OS) 1.5 vs. 10.7 months, HR = 1.00, 95%CI = 0.69–1.47). Nevertheless, the results confirmed the prognostic impact of CTCs: the median OS reached 35 months in patients with a low CTC count before treatment, 23 months in patients with a high CTC count before treatment but a low follow-up CTC count, and 13 months in patients with a persistently high CTC count. These differences were highly significant after adjusting for hormone receptor and HER2 status in a multivariable Cox model (*p* < 0.001). Overall, the study showed that for patients with persistently increased CTCs after one cycle of first-line chemotherapy, and that early switching to another standard chemotherapy regimen was not effective in terms of prolonged survival. Thus, persisting CTCs might represent a chemoresistant population of tumor cells which requires an alternative approach [15].

### 4.2. CirCe01

Another similar trial evaluating the response to chemotherapy by the CTC decrease after the first cycle is the CirCe01 study (NCT01349842). This French, multicenter, randomized phase III study included MBC patients that were CTC-positive (≥5 CTCs/7.5 mL) after two lines of chemotherapy. Patients were either followed by the conventional clinical and radiological assessments or by the determination of CTCs. Patients in the interventional (CTC-driven) arm were switched to another chemotherapy if CTC count did not decrease after one cycle of chemotherapy [16]. The aim was to switch non-responding patients early to avoid ineffective but toxic treatments. The recruitment has been completed, but the results are still pending.

### 4.3. STIC CTC

The French study group also initiated the STIC CTC Trial (NCT01710605). This phase III trial tries to answer the question of whether the choice between chemotherapy and endocrine therapy in first-line, HER2-negative, hormone receptor-positive MBC patients can be driven by the amount of CTCs. The patients were randomized to clinician’s choice in the standard arm (i.e., the decision for chemotherapy or endocrine therapy was based on clinical assessment) or CTC-based decision in the interventional arm. The trial used the common cutoff of ≥5 CTCs in 7.5 mL peripheral blood; patients in the CTC-driven interventional arm with less than 5 CTCs received endocrine treatment, while patients with 5 or more CTCs were treated with chemotherapy (by the physician’s choice). The results were presented at the San Antonio Breast Cancer Meeting 2018 [17]. Overall, 778 patients were randomized. In the clinician’s choice arm, 72.6% and 27.4% of patients respectively received hormone therapy and chemotherapy, while in the CTC-driven arm, 62.6% and 37.4% of patients were respectively treated with endocrine therapy and chemotherapy. The primary endpoint of the study was met, as PFS of patients in the CTC decision-based arm (median 15.6 months) was not inferior to PFS of patients in the clinician’s choice arm (median 14.0 months). The analysis focused further on the planned subgroup analysis of the discordant groups (CTCs high/clinical low risk; CTCs low/clinical high risk). Patients in the CTCs high/clinical low-risk subgroup who received a CTC-driven chemotherapy discordant to the physicians choice had a significantly longer PFS than the patients in the clinical-decision arm receiving endocrine therapy (HR = 0.62, 95%CI = 0.45–0.84; *p* = 0.002), with a non-significant trend toward longer OS (HR = 0.69, 95%CI = 0.43–1.11; *p* = 0.12). On the other side, patients in the CTCs low/clinical high-risk subgroup who were deescalated to a hormonal therapy from the clinically chosen chemotherapy due to having a low CTC count showed no significantly worse PFS compared to the patients treated with chemotherapy according to clinician’s choice. In addition, when the patients from the two discordant subgroups were pooled, patients receiving chemotherapy showed significantly better PFS (HR = 0.66, 95%CI = 0.51–0.85; *p* = 0.001) and OS (HR = 0.65, 95%CI = 0.43–0.98; *p* = 0.04) than patients receiving endocrine therapy, challenging current treatment standards. The results of this trial are promising and indicate that including a CTC count in the decision algorithm for HER2-negative, hormone receptor-positive MBC patients might improve patient outcome in some cases. However, the results need to be confirmed for the new era of endocrine treatment regimen with CDK4/6 inhibitors.

### 4.4. CirCe T-DM1

The CirCe T-DM1 study (NCT01975142) is the first clinical trial not using the number but the phenotype of CTCs as a decision criterion. HER2-negative MBC patients needing a third or fourth line of therapy and with detected HER2-positive CTCs in baseline-screening were treated with the antibody–drug conjugate trastuzumab emtansine (TDM-1). The study was closed after the first interim analysis and presented at the ESMO 2017 [18]. A total of 155 patients were screened, 14 (9.0%) had HER2-positive CTCs and 11 patients were treated with TDM-1. Partial response was observed in only one patient; median PFS was 4.9 months (range: 1.8–10.1). Due to these results and the very low prevalence of HER2-positive CTCs (1.6% of the detected CTCs), the authors conclude that the tested therapeutic approach was not promising.

### 4.5. DETECT Study Program

The DETECT study concept is a comprehensive trial observing CTCs for liquid biopsy in MBC patients with different biologic tumor features. The study concept includes DETECT III, DETECT IV, and DETECT V. It investigates the efficacy of treatment decisions based on the presence and phenotype of CTCs. 

Patients with HER2-negative MBC and HER2-positive CTCs are eligible for the DETECT III study (NCT01619111). In this phase III trial, 120 patients were randomized (1:1) to a standard endocrine (letrozole, anastrozole, or exemestane) or standard chemotherapeutic (docetaxel, paclitaxel, capecitabine, vinorelbine) treatment according to the physician’s choice, with or without an additional HER2-targeted treatment with lapatinib. The primary endpoint of the DETECT III study is the efficacy as assessed by the CTC clearance rate, i.e., the proportion of patients with no evidence of CTCs in the blood after treatment. 

Patients with HER2-negative MBC and only HER2-negative CTCs are being treated in the DETECT IV trial (NCT02035813), which is divided into two separate treatment and observation cohorts. In cohort A, patients with hormone receptor-positive breast cancer are treated with endocrine therapy (anastrozole, letrozole, exemestane, fulvestrant or tamoxifen; tamoxifen only for patients in combination with ribociclib) combined with the mTOR inhibitor everolimus or (after an amendment) the CDK4/6 inhibitor ribociclib. Cohort B includes patients with either triple-negative breast cancer or patients with hormone receptor-positive breast cancer that need a more aggressive treatment. Here, patients receive the mitotic inhibitor eribulin (halichondrin B analogue) as treatment in single-arm observation. The primary endpoints for cohort A and B are efficacy as assessed by the CTC clearance rate and PFS, respectively.

With DETECT V/CHEVENDO (NCT02344472), the study concept also offers a therapeutic option for patients with HER2-positive and hormone receptor-positive breast cancer, even though interventions are not based on CTCs. In this two-arm randomized phase III trial, all patients are treated with the dual HER2-targeted therapy with pertuzumab and trastuzumab, and are randomized to either in combination with chemotherapy (docetaxel, paclitaxel, capecitabine, vinorelbine) or endocrine therapy (fulvestrant, tamoxifen, letrozole, anastrozole, or exemestane) combined with the CDK4/6 inhibitor ribociclib. A modified adverse event score and the “quality-adjusted time without symptoms and toxicity” (Q-TWiST) method are used for assessing safety, tolerability and quality of life. In fact, the DETECT V trail is the only part in which the therapeutic decision is not driven by the number or phenotype of CTCs. However, one aim of the DETECT V trial is the development of an “endocrine responsiveness score” (ERS), based on the estrogen receptor and *HER2* expression of CTCs, to derive a predictive tool for the hormone receptor-positive, HER2-positive disease. The first results coming from the COMETI-2 study suggest that high rates of estrogen receptor are associated with a better response to endocrine treatment, while high rates of *HER2* expression are associated with a worse response [19]. The general goal of the endocrine responsiveness score is to identify patients with a predicted good response to endocrine therapy to avoid unnecessary chemotherapeutic treatments, which are generally associated with more adverse events and decreased quality of life compared to endocrine therapies.

In all trials of the DETECT study concept, CTCs are measured repetitively during treatment to obtain data on CTC dynamics and their possible role as a tool for treatment monitoring and early response assessment. Furthermore, the DETECT study concept is accompanied by a comprehensive translational research project (“DETECT-CTC”) which is funded by the Deutsche Krebshilfe (German Cancer Aid). The main aim of DETECT-CTC is to apply innovative biomarkers and assays focusing on molecular characteristics of CTCs and circulating nucleic acids to analyze their potential function as a liquid biopsy tool for assessing the biological status of the advanced disease, and to determine their relevance for predicting treatment response and therapy monitoring in order to optimize treatment for patients with metastatic breast cancer. Various subprojects of DETECT-CTC focus on the molecular characterization of CTCs, circulating free DNA and microRNA in blood, the evaluation of DNA damage and repair markers on CTCs, the evaluation of the origins and molecular causes of resistance to endocrine therapy at the level of individual CTCs, the comparison of phenotypic expression of biomarkers between CTCs, disseminated tumor cells from bone marrow, primary tumor and metastases from the same patients, and the study of microevolution of resistant subclones in metastatic breast cancer through single-cell analysis of CTCs [20].

In a comprehensive analysis of a large cohort of DETECT screening patients with HER2-negative primary tumors, the rate of discordance in the HER2 phenotype between primary tumor and CTCs was assessed and, as such, a discordance might have far-reaching implications in terms of follow-up treatment and the addition of targeted therapies [21]. The analysis included data from 1123 patients with HER2-negative MBC, and CTC screening was performed on average 56 months after primary diagnosis. In blood samples from 711 (63.3%) of 1123 patients, at least one CTC was detected (median 7 CTCs). To assess the HER2 status of CTCs, cells were labeled with HER2 antibodies, and only CTCs with a strong IHC staining intensity for HER2 (IHC score 3+) were considered as HER2-positive. In 134 of the 711 CTC-positive patients, at least one HER2-positive CTC was detected, yielding a HER2 discordance between primary tumor and CTCs in 18.8% of patients. A multivariable logistic regression with presence of at least one HER2-positive CTC (yes vs. no) as binary response variable revealed that lobular tumor histology, positive hormone receptor status, younger age, and the presence of 5 or more CTCs significantly predicted discordance in HER2 phenotype between primary tumor and CTCs. The authors concluded that in view of the well-known tumor heterogeneity in MBC, CTC-based liquid biopsy might better reflect total tumor burden and heterogeneity of tumor biology than biopsy of a single metastatic site.

Interestingly, the rates of patients with HER2-positive CTCs seem to be lower in the French CirCe T-DM1 trial (9.0% of screened patients) as compared to the DETECT screening (18.8% of screened patients). Both trials used the CELLSEARCH^®^ System for the detection of CTCs, and HER2-positivity as a criterion for patient inclusion was defined as the presence of at least one HER2-positive CTC both for the CirCe T-DM1 and the DETECT III trial. However, HER2-positivity was assessed based on single-cell FISH-analysis in CirCe T-DM1 and by an immunohistochemical antibody staining procedure in DETECT. Another difference between CirCe T-DM1 and DETECT is the fact that MBC patients could be included in the DETECT III trial regardless of treatment line, while for the CirCe T-DM1 trial only patients starting a third- or fourth-line systemic therapy were eligible. In CirCe T-DM1, the prevalence rate of HER2-positive CTCs out of all detected CTCs in included patients was very low with a median of 1.6%, and the French group concluded that the low prevalence rate of HER2-positive CTCs was an important reason for the failure of the study [18]. Results of the DETECT III trial are still pending, and it will be interesting to see whether the rate of HER2-positive CTCs out of all detected CTCs will be as low as the rate observed in the CirCe T-DM1 study.

## 5. Conclusions

A general finding of all trials evaluating the clinical utility for CTCs described here is that CTCs are rare cells which are not present in every patient, particularly so in patients with EBC. Nevertheless, there is ample evidence that CTCs—once detected—are a strong prognostic factor for reduced survival. However, at present, it is not clear how this knowledge can be transferred to a prediction of therapy response and an improved clinical outcome. The STIC CTC trial showed the first data for a positive effect of a CTC-based decision in a subgroup with a high count of CTCs before starting treatment. Thus, CTCs might represent a helpful early treatment monitoring tool and can be used in situations with uncertain therapy response. The awaited results of the ongoing trials described here will hopefully provide much needed data that help to answer the question regarding the clinical utility of CTCs. Furthermore, the identification of potential targets for more individualized treatment options might improve the use of CTCs in clinical practice. 

## Figures and Tables

**Table 1:** Clinical trials with CTC-based treatment decisions.

Trail	Status	Enrollment	Condition	Intervention	Primary Endpoints
**Treat-CTC, NCT01548677** **(phase II)**	Closed after interim analysis	1317 enrolled 63 randomized (CTC-pos)	HER2-neg EBC with CTCs after CT	Trastuzumab iv 6 cycles vs. observation	CTC detection rate at week 18
**SWOG S0500, NCT00382018** **(phase III)**	Completed	595 enrolled; 123 randomized (persisting high CTC count)	CT-resistant, CTC-pos MBC	Early switch in therapy vs. treatment until progression	OS, PFS
**CirCe01, NCT01349842** **(phase III)**	Recruitment completed,	568 planed	CT-resistant, CTC-pos MBC	Early switch in therapy vs. treatment until progression	OS
**STIC-CTC, NCT01710605** **(phase III)**	Completed	778 randomized	HR-pos and HER2-neg MBC	Decision CT or ET by clinical choice vs. CTC count	PFS, economic value
**Circe TDM-1 NCT01975142** **(phase II)**	Closed after interim analysis	155 screened; 11 treated	HER2-neg MBC and HER2-pos CTCs	T-DM1	TRR
**DETECT III, NCT01619111** **(phase III)**	Recruiting	120 planned, up to date: 105	HER2-neg MBC and HER2-pos CTCs	Standard treatment vs. Standard treatment + lapatinib	CTC clearance
**DETECT IV, NCT02035813** **(phase II)**	Recruiting	Group A: 180 planned Up to date: 103 Group B: 120 planned Up to date: 107	HER2-neg MBC and HER2-neg CTCs	A: ET + ribociclib or everolimus B: eribulin	A: CTC clearance B: PFS
**DETECT V, NCT02344472** **(phase III)**	Recruiting	270 planned Up to date 151	HR-pos, HER2-pos, MBC	trastuzumab/pertuzumab + CT or ET with ribociclib	Tolerability, safety, and quality of life

CT = chemotherapy, CTC = circulating tumor cells, ET = endocrine treatment, HER2 = human epidermal growth factor receptor 2, HR = hormone receptor, EBC = early breast cancer, MBC = metastatic breast cancer, OS = overall survival, PFS = progression-free survival, TRR = tumor response rate, T-DM1 = trastuzumab emtansine, pos = positive, neg = negative
